# Supplementary material for: Genetic Characterization of Carbapenem-Resistant Klebsiella spp. from Municipal and Slaughterhouse Wastewater
Source: Antibiotics (Basel). 2022 Mar 24;11(4):435. doi: 10.3390/antibiotics11040435 (PMC9027467; doi:10.3390/antibiotics11040435)
Supplement: Supplementary file 1 [file antibiotics-11-00435-s001.zip › antibiotics-1629332-supplementary.pdf]

Supplementary Material

Genetic characterization of carbapenem-resistant *Klebsiella* spp. from municipal and slaughterhouse wastewater

Mykhailo Savin, Gabriele Bierbaum, Nico T. Mutters, Ricarda Maria Schmithausen, Judith Kreyenschmidt, Isidro García-Meniño, Silvia Schmoger, Annemarie Käsbohrer and Jens Andre Hammerl

Corresponding authors:

Dr. Mykhailo Savin, Institute for Hygiene and Public Health, University Hospital Bonn,

Bonn, Venusberg-Campus 1, Building 63, 53127 Bonn, Germany

E-mail: m.savin@uni-bonn.de

Dr. Jens Andre Hammerl, Unit Epidemiology, Zoonoses and Antimicrobial Resistances,

Department of Biological Safety, German Federal Institute for Risk Assessment, Max-Dohrn

Str. 8-10, D-10589 Berlin, Germany

E-mail: jens-andre.hammerl@bfr.bund.de

Table S1: Mobile genetic elements detected in carbapenem-resistant *Klebsiella* spp. isolates recovered from municipal WWTPs and their receiving water bodies as well as from process waters of poultry and pig slaughterhouses.

***Klebsiella* isolate ID**	**Plasmid replicons detected**	**Plasmid-associated AMR**	**IS without AMR determinants**	**IS-associated with AMR determinants**
05/10-60	IncR	*aph*(3")-Ib-*aph*(6)-Id (IncR)	IS6100, IS26	*sul2* (ISVsa3)
03/11-38	IncFII(Yp), Col440I, IncA/C, IncFIB(K)	n.d.	ISEcl1, ISSen4, IS30, ISEsa2, IS903, cn_4959_IS903, ISEc33, IS102	*bla*$_{SHV-12}$ (ISAhy2)
05/10-83	IncFIB(K), IncFII(K), IncFIB(pKPHS1), Col440I	n.d.	IS903, Tn5403, ISEc31, IS102, ISEcl1, ISKpn1, IS5075	*bla*$_{TEM-1B}$-*sul2*-*aph*(6)-Id-*aph*(3")-Ib-*bla*$_{CTX-M-15}$ (ISEc9), *qnrB1*-*tet*(A) (Tn5403), *dfrA14* (IS6100)
05/11-32	IncR	*aph*(3")-Ib-*aph*(6)-Id (IncR)	IS6100, IS26	*sul2* (ISVsa3)
05/11-43	IncFIB(K), Col440I, IncFIB(pKPHS1)	n.d.	ISKox1, ISKpn42, ISSty2, ISEc31, ISKpn1, ISEcl1, ISSen4, IS5075, ISKpn26	*bla*$_{TEM-1B}$-*sul2*-*aph*(6)-Id-*aph*(3")-Ib-*bla*$_{CTX-M-15}$ (ISEc9)
05/13-25	IncR	*aph*(3")-Ib-*aph*(6)-Id (IncR)	IS6100, IS26	*sul2* (ISVsa3)
05/10-58	IncR	*aph*(3")-Ib-*aph*(6)-Id (IncR)	IS6100, IS26	n.d.
05/11-29	IncFII(K), IncFIB(K), Col440II	n.d.	ISKpn14, cn_5267_ISKpn14, ICEEcoED1a-1, ISKpn33, ISKpn26, ISEc9, ISEcl1	n.d.
03/13-21	Col440I, IncFII(K))	*bla*$_{CTX-M-15}$ (IncFII(K))	ISEc9, ISSen4, IS26	*dfrA14* (IS6100)
04/08-35	IncR, ColpVC, IncN	n.d.	ISKpn1	

01/07-41	IncX4, IncFIB(pKPHS1)	n.d.	ISEch12, IS26	*qnrS1*-*bla*$_{LAP-2}$ (ISKpn19), *qacE*-*sul1*-*dfrA1* (IS6100)
05/10-21	IncFIB(K), IncFII(K), IncHI2A, IncHI2, Col440I	n.d.	ISKpn14, ISKpn34, ISKpn43, ISKpn28, ISEsa2, ISEsa1, Tn6196, ISKpn8, IS5075	n.d.
05/10-59	IncR, IncFIA(HI1)	n.d.	ICEEcoED1a-1, ISKpn26, ISKpn38, ISEsa2, ISEsa1, ISKpn1, IS5075, ISKpn28	*dfrA14* (IS6100), *bla*$_{CTX-M-15}$ (ISEc9)
03/05-22	IncFIB(K), IncFII, IncFIA(HI1), IncHI1A, IncHI1B(R27), ColRNAI, IncFII	*sul2*-*aph*(6)-Id-*aph*(3")-Ib (IncQ1)	IS629	*mph*(A) (IS6100), *floR* (ISVsa3), *tet*(B) (ISVsa5)
03/10-46	IncFIB(K)	n.d.	ISEam1	*qnrS1*-*aph*(6)-Id-*aph*(3")-Ib-*sul2* (ISKpn19), *mph*(A)-*qacE*-*sul1*-*aadA2*-*dfrA12* (IS6100), *bla*$_{CTX-M-15}$ (ISEc9)
05/11-30	IncR	n.d.	ISKpn26, IS6100, IS26	*sul2* (ISVsa3)
03/12-04Bki	IncHI2, IncHI2A, IncR	n.d.	IS6100, ISKpn21, ISEsa2, ISEsa1, ISKpn26, cn_9071_ISKpn21, Tn6196, ISKpn8, IS102, IS26	n.d.
03/11-12	Col440I, IncFIB(K), IncR, Col(IRGK), Col(IMGS31), IncFIB(pKPHS1)	n.d.	ISEc33, ISKpn21, ISPpu12, ISApu1, cn_3415_ISApu1, ISEcl1, ISAs2, ISKpn8, IS903, ISAeme4, ISSpu2, ISKpn28	n.d.

05/10-21B	IncHI2, IncHI2A, IncFIB(K), Col440I, IncFII(K)	n.d.	ISKpn14, ISCfr26, ISKpn43, ISKpn34, ISKpn28, ISEsa2, ISEsa1, Tn6196, ISKpn1, ISKpn8, IS5075	n.d.
05/13-23	IncHI1B	*tet*(A) (IncFIB(Mar))	ISKpn38, IS5075, IS26	*qnrS1*-*aph*(6)-Id-*aph*(3")-Ib-*sul2* (ISKpn19), *mph*(A) (IS6100), *bla*$_{CTX\text{-}M\text{-}15}$ (ISEc9)
03/01-52	IncFIB(K), IncFII, ColRNAI, Col440II	n.d.	ISEch12, ISSty2,	*mchF* (GIE492)
05/10-69A	IncFIA(HI1), IncFIB(K), IncFII(K), Col440I,	n.d.	ISKpn14, ISPlge4, ISKpn14, ISEc33, ISEc31, ISKpn42, ISSen4, Tn5403, ISEc15, ISKpn21, IS5075, ISKpn26, IS903, IS102, ISKpn28	*bla*$_{TEM\text{-}1B}$ (Tn2)
01/07-40	IncFIB(pKPHS1), IncX4, Col440I	n.d.	n.d.	*qnrS1*-*bla*$_{LAP\text{-}2}$ (ISKpn19), *qacE*-*sul1*-*dfrA1* (IS6100)
03/06-23	IncFII, IncFIB(K), IncHI1A, IncHI1B(R27), IncFIA(HI1)	*sul2*-*aph*(6)-Id-*aph*(3")-Ib (IncQ1), *ccI* (ColRNAI)	IS629	*mph*(A) (IS6100), *floR* (ISVsa3), *tet*(B) (ISVsa5)
05/10-69B	IncFII(K), Col440I, IncFIA(HI1), IncFIB(K)	n.d.	ISKpn14, ISPlge4, ISEc33, ISEc31, ISKpn42, ISSen4, Tn5403, ISEc15, ISKpn21, IS102, IS5075, ISKpn26, ISKpn28	*bla*$_{TEM\text{-}1B}$ (Tn2)
05/10-20A	Col440I, IncHI2, IncHI2A, IncFIB(K), IncFII(K)	n.d.	ISKpn14, ISCfr26, ISKpn43, ISKpn34,	n.d.

			ISKpn28, ISEsa2, ISEsa1, Tn6196, ISKpn8, IS5075	
05/10-71	IncFIB(K), Col440I, IncFII(K)	n.d.	ISKpn42, ISSen4, ISKpn14, ISPlge4, IS5075, ISEc33, ISEc31, Tn5403, ISKpn21, ISKpn26, ISKpn28	n.d.
05/13-31	n.d.	n.d.	n.d.	*dfrA14* (IS6100), *bla*$_{CTX-M-15}$ (ISEc9)
05/10-20B	IncFIB(K), IncFII(K), Col440I, IncHI2, IncHI2A	n.d.	ISKpn14, ISCfr26, ISKpn43, ISKpn34, ISKpn28, ISEsa2, ISEsa1, Tn6196, ISKpn1, ISKpn8, IS5075	n.d.
03/11-28	IncFIB(K)	n.d.	ICEEcoED1a, IS903, ISEc15, IS102, ISEcl1, IS5075	*dfrA14* (IS6100)

n.d., not detected; IS, insertion sequence; cn, composite transposon; Tn, transposon; ISE, insertion sequence element; ICE, Integrative Conjugative Element
